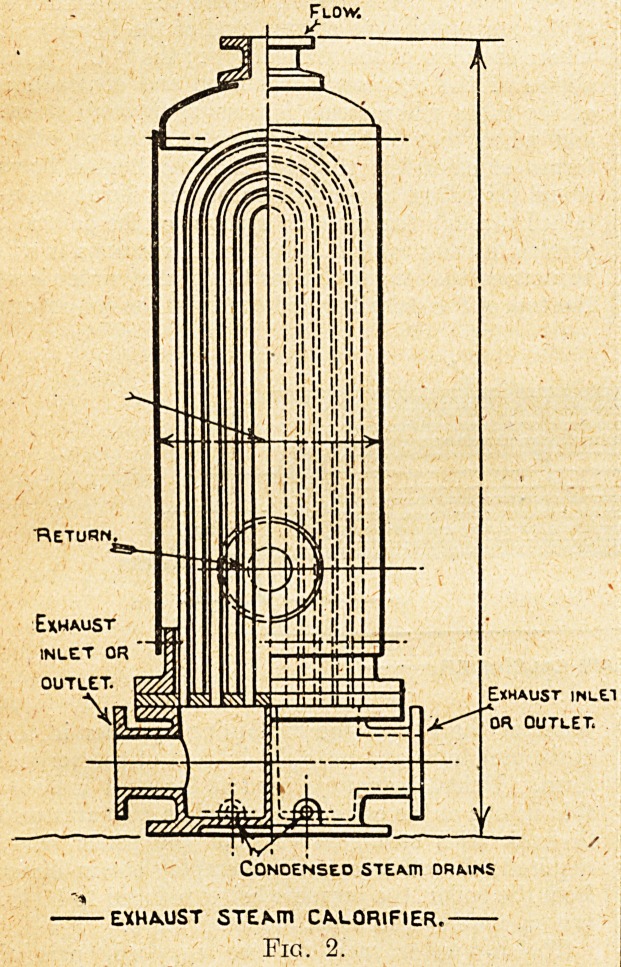# Middlesex Hospital

**Published:** 1918-01-19

**Authors:** 


### THE HEATING OF HOSPITALS.

### XIII. Middlesex Hospital.

### THE USE OF WATER-TUBE BOILERS FOR THE PROVISION OF STEAM FOR RADIATORS.

As mentioned in a previous article, not many hospitals, comparatively speaking, employ water-tube boilers, though the number is apparently creeping up. There are already fifteen hospitals, asylums, and nursing homes in which Babcock and Wilcox boilers are employed in the United Kingdom, and a fairly large number in our Dominions and in foreign countries. The "tank" boiler, as the Americans call it, the Lancashire boiler, or the Cornish boiler as we call it, has been found more suitable for the requirements of hospitals because of its steady steaming qualities; it may be depended upon to keep steam right throughout the twenty-four hours, and to answer any sudden demand at any moment of the day or night. There are cases, however, and the Middlesex Hospital is one of them, where the water-tube boiler has to be employed, if steam is to be used at all, because there is no means of getting the large "tank" boiler into the position where it is required. In the case of the Middlesex Hospital about eleven years ago it was necessary to provide increased boiler power, and as the hospital building is shut in everywhere by other buildings it would have necessitated extensive structural alterations to those buildings to enable a large Lancashire boiler to be put in position. As the water-tube boiler can be separated into a number of comparatively small pieces the difficulty was overcome. The writer understands that, at the same time as the Babcock and Wilcox water-tube boilers were put in, the opportunity was taken to concentrate the heating plant at one convenient centre, as has been done in so many other hospitals. The two boilers, each capable of evaporating 7,000 lb. of water per hour, and converting it into steam at 100 lb. pressure per sq. inch, replaced nine boilers of various types, some of them the old vertical boilers, some of them hot-water boilers, and some of the marine type. The steam from the water-tube boiler is employed for driving various pumps and engines in different parts of the hospital; it furnishes steam for the absorption refrigeration plant, and it is used for heating the water for domestic use, and for hot-water radiators.

Calorifiers, of which two forms are shown in figs. 1 and 2, supplied by the Wright Forge and Engineering Company, of Tipton, Staffs, are employed to heat the water for both purposes. The construction of the calorifiers is shown in the illustrations; there is the usual containing cylinder, and the pipes through which the steam flows fixed on the inside of the cylinder. The pipes are of the "U" form, their ends being expanded into plates at one end of the cylinder, where the calorifier is of the horizontal type shown, and at the lower end, where they are of the vertical type; the "U" form allows of the necessary expansion and contraction of the tubes when steam is passing through them, and when they are out of service. As in other forms of calorifier, the water to be heated flows round the tubes, a continuous current being maintained through the radiator system, or the domestic service heating system. In the illustrations it will be noticed that the outlet for the heated water is at the top of the calorifier and the inlet for the return water at the end of the containing cylinder in the horizontal form and near the bottom of the vertical form. Exhaust steam is used as far as it is available in one of the calorifiers, which is also arranged to work with steam direct from the boiler when required; the other calorifiers employ steam direct from the boiler, the pressure being reduced to 50 lb. per sq. inch by the usual reducing valve.

Fig. 1.

The hot-water radiators are supplied, part of them on the thermo-syphon system, in which the flow of water is maintained entirely by the difference in weight between the hot column flowing upwards and the return column flowing downwards, and partly on the accelerated system that has been described in connection with other hospitals. It will be remembered that in the accelerated system either a pump or what is called an impeller, practically a revolving disc with vanes, is placed in the path of the water. In the accelerated system the pipes delivering the water to the radiators can be of much smaller diameter than with the thermo-syphon system. At the Middlesex Hospital the heating plant has developed, as in so many other hospitals, from the open-fire method, which is still employed to a certain extent, to the water radiator

**THE HEATING OF HOSPITALS**—(*continued*).

by thermo-syphon method, and to the accelerated system.

A point that is of considerable importance in connection with the heating of hospitals is the pressure at which the steam in the boiler is generated. There is a tendency steadily to increase the pressure, and the use of the water-tube boiler tends to accentuate this. As mentioned in former articles, the water-tube boiler was originally introduced to enable higher steam pressures to be used than was possible with the tank boilers of those days owing to the quality of the material from which boilers were made and the tools at the disposal of the

EXHAUST STEAM CALORIFIER.

FIG. 2.

boiler makers. That time has passed; tank boilers to-day can carry pressures just as high as water-tube boilers can carry. The higher pressures, however, were introduced distinctly in the service of the power engineer; it is of great service where steam is used for power to raise it to as high a pressure as possible, and to condense it at as high a vacuum as possible after it has done its work in the engine or turbine that is furnishing power; but the case is quite different where heat only is required from the steam, or where the power makes only a comparatively small demand upon the total quantity of steam raised; and this, as the writer understands the matter, is the case with the large majority of hospitals. Steam is a very useful agent for carrying heat to a distance, but it does not require to be high-pressure steam; and there is a distinct disadvantage in using higher pressures than are necessary to ensure the transport of the steam to the farthest point at which heat is required. For this purpose pressures in the neighbourhood of 60 lb. per sq. inch are quite sufficient. When the question of delivering the heat carried by the steam to the water or to the air has to be considered, the lower the pressure that can be employed the better. When steam is formed by the evaporation of water the major portion of the heat, nearly 1,000 B.Th. units per lb., is absorbed in the conversion of the water into steam; and when the steam condenses in the calorifier, the steam radiator, or in one of the numerous devices that have been introduced for the purpose, it gives up the whole of this heat to the objects near it; and it depends upon the efficiency of the apparatus how much is absorbed by the water or the air to be heated. Very little more heat is delivered to water or air by high-pressure steam than by low-pressure: only a few more B.Th. units per lb.; but the presence of the higher pressure tends to cause increased condensation of the steam where it will do no useful work, as in the pipes leading to the calorifiers, the radiators, etc., and it tends to cause increased leakage of the steam through imperfect joints. High-pressure steam also requires that the whole apparatus using it shall be of greater mechanical strength than low-pressure steam, and therefore it is more expensive to manufacture.

## Figures and Tables

**Figure f1:**
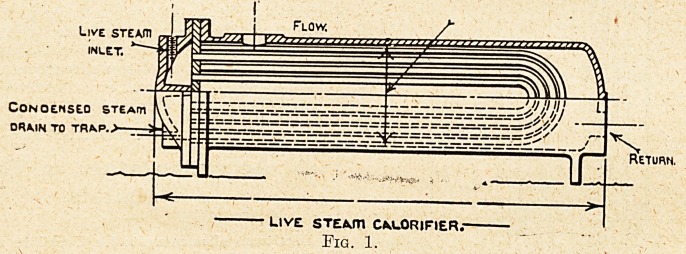


**Figure f2:**